# Clustering patients with late-life depression by blood glutathione-dependent enzymatic activities for stratification of a heterogeneous group

**DOI:** 10.1192/j.eurpsy.2024.1335

**Published:** 2024-08-27

**Authors:** T. Prokhorova, I. Boksha, O. Savushkina, E. Tereshkina, E. Vorobyeva, G. Burbaeva

**Affiliations:** FSBSI “MENTAL HEALTH RESEARCH CENTRE”, Moscow, Russian Federation

## Abstract

**Introduction:**

We have previously found significant alterations in activities of glutathione dependent enzymes in blood cells of patients with late-life depression (LLD) compared with age-matched controls.

**Objectives:**

The revealing subgroups of LLD patients by glutathione-metabolism enzymes’ activities in blood cells using cluster analysis.

**Methods:**

LLD patients (n=101) of 60-86 age (69 patients with recurrent depression (RD), 23 with bipolar disorder (BD) and 9 patients with a single depressive episode (DE)) were assessed by Hamilton depression rating scale (HAMD-17), and Hamilton Anxiety Rating Scale (HARS). Activity levels of glutathione reductase (GR) and glutathione S-transferase (GST) were determined in patients’ platelets (-pl) and erythrocytes (-er). The control group consisted of 51 peoples 55-84 years old without mental pathology. Cluster analysis module of the STATISTICA software was used for clustering the patients by baseline blood parameters.

**Results:**

Three clusters of patients were obtained: C1, n=39, C2, n=31, C3, n=31, differing significantly in all biochemical parameters (Kruskal-Wallis test, *p*<0.001), except GST. When compared with control group by Mann-Whitney test, GST-pl, GST-er, and GR-er were significantly decreased in C1; GST-er was significantly increased in C2; GST-pl, GR-pl, and GR-er were significantly decreased in C3. Several significant correlations were found between the measured parameters and scores by HDRS or HAMD-17. In C1, baseline activity of GST-er correlated with total scores by HAMD-17 (R=0.335, p=0.043) after treatment. In C2, baseline activity of GR-er correlated with total scores by HARS (R=-0.376, p=0,037) after treatment and GR-pl correlated with delta scores by HAMD-17 under the treatment (R=0.484, p=0.006). No significant correlations were found in C3. Patients with BD distributed significantly unevenly between C1, C2, and C3, with significantly more BD patients clustering in C1 (61%) compared with C2 and C3 (Yetes-corrected Chi-square =7.73, p=0.0054), whereas patients with RD and DE distributed evenly.

**Conclusions:**

Patterns of activity levels for glutathione-dependent enzymes in patients with BD differ from those in patients with RD and DE. Significant correlations of the measured biochemical parameters with scores by HDRS or HAMD-17 assessed after the treatment and evidenced for the treatment efficacy seem to be promising biomarkers for further evaluation of the treatment efficacy in heterogeneous group of LLD patients using the proposed approach to their stratification into subgroups.

**Disclosure of Interest:**

None Declared

